# A performance study of the impact of recombination on species tree analysis

**DOI:** 10.1186/s12864-016-3104-5

**Published:** 2016-11-11

**Authors:** Zhiwei Wang, Kevin J. Liu

**Affiliations:** Department of Computer Science and Engineering, Michigan State University, 428 S. Shaw Lane, East Lansing, 48824 USA

**Keywords:** Phylogenetic, Phylogenomic, Species tree inference, Recombination, Linkage disequilibrium, Recombination breakpoint

## Abstract

**Background:**

The most widely used state-of-the-art methods for reconstructing species phylogenies from genomic sequence data assume that sampled loci are identically and independently distributed. In principle, free recombination between loci and a lack of intra-locus recombination are necessary to satisfy this assumption. Few studies have quantified the practical impact of recombination on species tree inference methods, and even fewer have used genomic sequence data for this purpose. One prominent exception is the 2012 study of Lanier and Knowles. A main finding from the study was that species tree inference methods are relatively robust to intra-locus recombination, assuming free recombination between loci. The latter assumption means that the open question regarding the impact of recombination on species tree analysis is not fully resolved.

**Results:**

The goal of this study is to further investigate this open question. Using simulations based upon the multi-species coalescent-with-recombination model as well as empirical datasets, we compared common pipeline-based techniques for inferring species phylogenies. The simulation conditions included a range of dataset sizes and several choices for recombination rate which was either uniform across loci or incorporated recombination hotspots. We found that pipelines which explicitly utilize inferred recombination breakpoints to delineate recombination-free intervals result in greater accuracy compared to widely used alternatives that preprocess sequences based upon linkage disequilibrium decay. Furthermore, the use of a relatively simple approach for recombination breakpoint inference does not degrade the accuracy of downstream species tree inference compared to more accurate alternatives.

**Conclusions:**

Our findings clarify the impact of recombination upon current phylogenomic pipelines for species tree inference. Pipeline-based approaches which utilize inferred recombination breakpoints to densely sample loci across genomic sequences can tolerate intra-locus recombination and violations of the assumption of free recombination between loci.

**Electronic supplementary material:**

The online version of this article (doi:10.1186/s12864-016-3104-5) contains supplementary material, which is available to authorized users.

## Background

Recombination is pervasive throughout the eukaryotic Tree of Life [1], and modeling and methodological development to enable recombination-aware phylogenetic inference has been an active area of study. Traditional methods utilize non-parametric and parametric approaches to account for point mutations and recombination. More recently, phylogenomic modeling and inference methods account for heterogeneous evolutionary processes that result in local patterns of genealogical variation, including recombination, point mutations, genetic drift, and the complex interplay of these processes acting in combination. The primary models utilized for this purpose are based upon the coalescent-with-recombination (CWR) model [2, 3]. The models find application in a variety of inference and learning tasks throughout populations genetics, phylogenetics, and phylogenomics [1]. Approximations to the CWR model such as the sequentially Markovian coalescent model [4] enable greater scalability for inference and learning. These modeling and methodological advances have rekindled interest in the phylogenomic study of recombination. In an influential review, Edwards [1] posits that recombination has a major impact on species phylogeny inference, and methodological work to account for recombination should therefore play a prominent role in phylogenetics and phylogenomics. Since then, only a few studies have attempted to directly quantify the impact of recombination on state-of-the-art phylogenomic inference methods. Recently, Lanier and Knowles conducted a simulation study to investigate this question [5]. In their study, each 8-taxon simulation sampled at most 9 identically and independently distributed (i.i.d.) loci from a multi-species CWR model. The study focused on performance comparisons using STEM [6], a maximum likelihood-based method for species tree inference given an input set of gene trees, and *BEAST [7], a Bayesian method that performs simultaneous inference of a species tree and gene trees under the multi-species coalescent model. One of the main conclusions was that violations of the assumption of zero intra-locus recombination was of secondary concern in terms of species tree inference accuracy, assuming free recombination between loci. Here, a practical issue has been noted by [8] and others. Outside of a simulation study, the theoretical distribution is not accessible for sampling i.i.d. loci. Even assuming a particular distribution is applicable, only the sequence data are observed, not the ancestral recombination graph and sequence breakpoints induced by historical recombination events. It is therefore premature to draw conclusions about the impact of recombination upon species tree inference accuracy. There are two sides to the i.i.d. assumption: no intra-locus recombination, and free recombination between loci. (In our study, a locus is the position of a DNA sequence on a chromosome, where the sequence may or may not correspond to a gene or other genomic feature. Similarly, a gene tree is the phylogeny of a single locus.)

As a practical matter, a variety of techniques are used to satisfy the assumption of free recombination between loci (with hopefully little or no recombination within each locus). The techniques are broadly categorized by whether they are data-driven or not. One data-driven technique preprocesses sequences using calculations based upon measures of linkage disequilibrium (LD). This approach, which we refer to as LD-based preprocessing, samples loci sufficiently far apart so that enough recombination events have occurred to ensure linkage equilibrium. In practice, this distance is determined by measuring LD between pairs of sites, and then examining LD decay as the distance between sites increases. As the distance increases, LD decay slows and observed LD converges to a background equilibrium level. An empirical cutoff is assessed to be the distance at which LD decay converges, and loci are sampled at an interval equal to the cutoff. Another data-driven technique explicitly infers recombination breakpoints, and each putatively recombination-free interval between a pair of neighboring breakpoints serves as a locus. A wide variety of parametric and non-parametric techniques have been proposed to infer recombination breakpoints along DNA sequences (reviewed by [9] and [8]). Among the simplest of these are techniques that utilize the Four-Gamete Test [10] (FGT) which requires the restrictive assumption of evolution under the infinite sites model. Other alternatives which are not data-driven include the use of gene annotations as loci without regard to ancestral recombination, sliding window approaches, and others.

In this study, we revisit the larger question captured by the title of the Lanier and Knowles study: “is recombination a problem for species tree analysis”? We focus in particular on the major open question regarding widely used phylogenomic inference pipelines and their use of various techniques to satisfy the assumption of free recombination between loci.

## Methods

Our study utilized simulated and empirical datasets to evaluate the impact of recombination on different phylogenomic inference pipelines. Here, we describe the methods used in our study. (Specific commands and software options are given in Additional file 1.)

### Simulation study

Simulations under the coalescent model with uniform recombination rate across loci followed the general protocol in [5]. Species trees with 8, 15, and 25 taxa were generated under a uniform speciation model using Mesquite [11]. To further validate our findings, we also included alternative model trees which consisted of the 10 8-taxon model trees from the simulation study of [5] and an empirical species tree based upon the consensus *Mus* phylogeny reported by Guénet and Bonhomme (see Fig. 1 in [12]). The former can be downloaded as part of the supplementary data provided in [5], and the latter is listed in Additional file 1. For each model condition, 20 replicates were generated. Each species tree had a total depth of 1N. For each species tree, coalescent gene trees were generated by ms [13] under the multi-species coalescent with a finite-sites model of recombination. We used 3 different choices for the population recombination rate *ρ*: 100, 200, and 1000. For the simulated sequence length used in our study (10 Mb) and effective population size of 2500, a *ρ* value of 1000 corresponds to a per-generation crossover probability between adjacent sites of 10^−8^. These values are within the range of estimates for mouse, rat, and human [14] (e.g., an empirical study of human demography estimated a population recombination rate of 13560 for use in related simulations involving a finite-sites model of recombination and sequence length of 30 Mb [15].) Sequence evolution was then simulated using the resulting gene trees as input. We used Seq-Gen [16] to simulate DNA sequence evolution under an HKY85 +*Γ* substitution model with *α*=0.8. The simulated sequence length for each replicate dataset was 10 Mb.

For each replicate, we ran four different phylogenomic inference pipelines. The pipelines differed based upon the set of loci and gene trees used as input to species tree analysis, where one of the following five options were used:

- **The LD-based sequence preprocessing approach discussed above with locus length of 1000 bp, which we refer to as “LD1000”.** For each sequence alignment, we estimated an empirical cutoff based on the LD decay plot using *r* ^2^ to measure LD (equation 7.13 in [3]). (See Additional file 1 for LD decay plots and empirical cutoffs.) Loci were then sampled at an interval equal to the empirical cutoff. The sequence length of each sampled locus was 1000 bp, which was identical to the locus length used by [5]. FastTree [17, 18] was used to infer a gene tree on each locus under the GTR +*Γ* substitution model.
- **The LD-based sequence preprocessing approach discussed above with locus length of 100 bp, which we refer to as “LD100”.** The LD100 method was otherwise identical to the LD1000 method.
- **An inferred breakpoints/inferred gene trees approach, which we refer to as “IBIG”.** The sequence was partitioned into blocks using the LRScan algorithm [19, 20] with each block satisfying the Four-Gamete test to rule out historical recombination [10]. We used a custom implementation of the LRScan algorithm which is provided as open-source software at the URL given in Additional file 1. To reduce computational burden at the potential expense of downstream phylogenomic inference accuracy, we chose to concatenate every 1000 blocks into a single locus, rather than letting each block correspond to a locus for the purpose of phylogenomic inference. (See Additional file 1 for an experiment that explores different settings for the concatenation step.) For this reason as well as the simple FGT-based approach, IBIG’s accuracy can be interpreted as a lower bound on the accuracy of phylogenomic pipelines which incorporate explicit breakpoint analysis. The lower bound suffices for the purposes of our study. (Recall also the findings of [5], which suggest that state-of-the-art phylogenomic inference pipelines are largely robust to violations of the assumption of zero intra-locus recombination.) A gene tree was then estimated on each locus using FastTree, similar to the above methods.
- **A true breakpoints/inferred gene trees approach, which we refer to as “TBIG”.** This approach made use of the true recombination breakpoints. Each recombination-free interval between a pair of neighboring breakpoints served as a locus in downstream analyses. Gene trees were inferred on loci using FastTree [17, 18], similar to the above methods.
- **A true breakpoints/true gene trees approach, which we refer to as “TBTG”.** This approach used the set of true gene trees (and, implicitly, the set of true recombination breakpoints) for each replicate dataset as input for downstream analysis.

The main motivation behind the use of ground truth in the TBIG and TBTG methods was for theoretical comparison with the other methods, which did not make use of ground truth. Thus, the accuracy of TBIG and TBTG serves to bound the potential accuracy of the other methods.

Given a set of gene trees inferred using one of the four approaches described above, each pipeline utilized ASTRAL-II [21, 22] to perform species tree inference. Our choice was motivated by prior studies which have shown ASTRAL-II to be among the most accurate state-of-the-art methods while offering much improved computational efficiency [21, 22].

As an alternative to the modeling assumption of uniform rate of recombination across loci, we also used the msHOT simulation tool [23] to perform coalescent simulations incorporating recombination hotspots. The simulations utilized the 8-taxon species trees that we generated using Mesquite. The recombination hotspots were simulated using two different approaches:

- **The procedure used by [** 24 **], where the number and length of hotspot regions were chosen deterministically.** The locations of 10 hotspots were chosen uniformly at random within an alignment. The 10 hotspot lengths were: two hotspots with length 1 kb each, two with 2 kb length, two with 3 kb length, two with 4 kb length, and two with 5 kb length. Each hotspot had local recombination rate that was 10 times the background recombination rate used outside of hotspots.
- **The procedure used by [** 25 **], where the number and length of hotspot regions were chosen non-deterministically.** The number of hotspots was drawn from a Poisson distribution parameterized so that the average distance separating neighboring hotspots was 500 kb. The width of each hotspot (in kb) was draw uniformly in the open interval (1,2). The intensity above background for each hotspot was drawn uniformly from the open interval (1,10).

In both approaches, the background recombination rate was 100. Otherwise, simulations incorporating recombination hotspots utilized a procedure that was identical to simulations elsewhere in our study: msHOT was used to simulate gene trees and locus lengths, and seq-gen was then used to simulate sequence evolution using the procedure described above.

For each dataset, the topological distance between an estimated species tree and the true species tree was measured using normalized Robinson-Foulds (RF) distance [26]. We used a routine implemented in the PhyloNet software package for this purpose [27].

### Empirical study

Our empirical study utilized genomic sequence data from a previous phylogenomic study of house mouse and sister species [28]. The dataset contains SNPs sampled broadly from 19 chromosomes which were genotyped using the Mouse Diversity Array [29]. The array’s SNP coverage was designed to be well-suited for understanding house mouse diversity and phylogenetics [29]. We used phased haploid sequences from 58 samples representing 8 different mouse species. (See [28] for details about genotyping and phasing.)

The IBIG and LD-based pipelines were used to infer species phylogenies on the empirical dataset. The LD-based approach was adapted to explore the impact of locus length on downstream phylogenomic inference. We therefore included between 1 and 15 neighboring SNPs in each sampled locus used in LD-based pipeline analyses. As in the simulation study, FastTree was used to infer a gene tree on each locus, and ASTRAL-II was used to infer a species tree given a set of gene trees as input.

## Results

### Simulation study

**Fig. 1:**
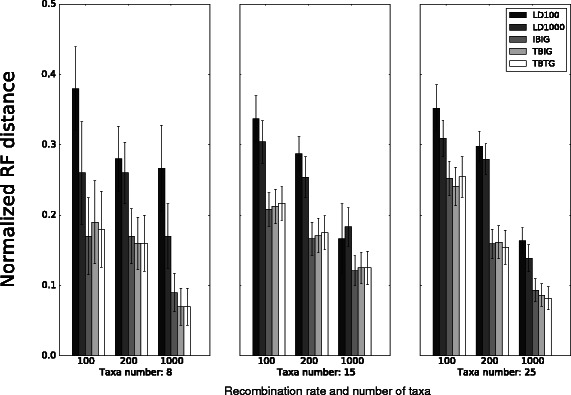
Topological accuracy of LD-based and breakpoint-based methods on simulated datasets. The model conditions had dataset size ranging from 8 to 25 taxa and recombination rate ranging from 100 to 1000, which was uniform across loci. Topological accuracy of each method was measured using the RF distance between the inferred and model phylogenies [26]. Each of the three breakpoint-based methods utilized one of the following inputs: inferred breakpoints/inferred gene trees (“IBIG”), true breakpoints/inferred gene trees (“TBIG”), or true breakpoints/true gene trees (“TBTG”). Averages and standard error bars are shown (*n*=20)

We began by comparing the topological accuracy of the LD-based and breakpoint-based methods on model conditions which incorporated a range of recombination rates and dataset sizes. For the smallest dataset size and any of the recombination rates explored in our study, the LD-based methods consistently returned worse accuracy than any of the breakpoint-based methods, and LD100 had similar or reduced accuracy compared to LD1000 (Fig. 1). The breakpoint-based methods had similar topological accuracy, differing by at most 0.02 in terms of average normalized RF distance. Notably, IBIG did not make use of ground truth like TBIG and TBTG, and yet IBIG had comparable accuracy to the other breakpoint-based methods regardless of recombination rate. On the other model conditions which had larger dataset sizes, the LD-based methods were consistently less accurate than the breakpoint-based methods, and similar accuracy was obtained regardless of whether inferred or true recombination breakpoints and gene trees were used as part of a breakpoint-based pipeline analysis, differing by at most 0.013 in terms of average normalized RF distance. The difference in topological accuracy of LD1000 and LD100 was smaller on model conditions with 15 and 25 taxa as compared to model conditions involving 8 taxa. For a given recombination rate, the topological accuracy of each method was generally similar across the different dataset sizes in our study.

**Fig. 2:**
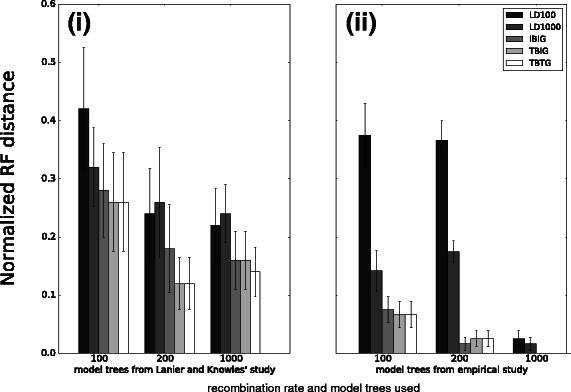
Topological accuracy of LD-based and breakpoint-based methods on datasets simulated using alternate model phylogenies. For each model phylogeny, coalescent simulation utilized a recombination rate ranging from 100 to 1000 which was uniform across loci. Topological accuracy of each method was measured using the RF distance between the inferred and model phylogenies [26]. In panel (*i*) on the left, results are shown for the set of 10 model phylogenies used in [5]. Following their study protocol, simulation was repeated for each model phylogeny to obtain 10 replicates, and averages and standard error bars are shown (*n*=10). In panel (*ii*) on the right, results are shown for simulations that utilized an empirical phylogeny. Averages and standard error bars are shown (*n*=20)

We also performed simulations using the model trees from the simulation study of Lanier and Knowles [5] as well as an empirical phylogeny. Results are shown in Fig. 2 panels (i) and (ii), respectively. For both simulations, the performance outcomes were consistent with the rest of our simulation study. The LD-based methods were less topologically accurate than the breakpoint-based methods across the different recombination rates explored in our study, and the performance advantage of LD1000 over LD100 was similar to our findings on the other 8-taxon model conditions. IBIG was either comparable in accuracy or slightly less accurate compared to the breakpoint-based methods that made use of true breakpoints and/or true gene trees. As the recombination rate increased, the topological accuracy of the different methods generally increased. For simulations involving the 10 8-taxon model trees from [5], two differences were observed compared to the rest of the simulation study: we observed generally greater topological error, and the difference in accuracy between the LD-based and breakpoint-based methods was smaller. For simulations involving the empirical phylogeny, two trends were observed which differed from elsewhere in the simulation study: the breakpoint-based methods had relatively lower error, and topological error on the model condition with the highest recombination rate was lower as well.

**Fig. 3:**
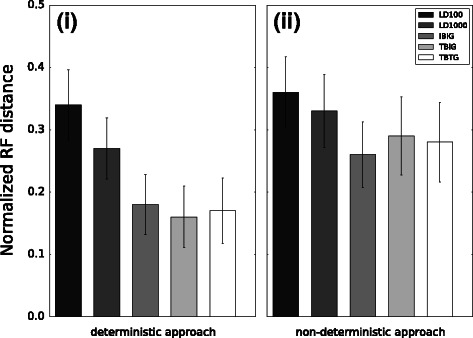
Topological accuracy of LD-based and breakpoint-based methods on model conditions with recombination hotspots. The number of recombination hotspots and the hotspot length distribution were chosen using either a deterministic or non-deterministic approach (see Methods). Results for 8 taxon simulations using the deterministic approach are shown in panel (*i*) on the left, and results for 8 taxon simulations using the non-deterministic approach are shown in panel (*ii*) on the right. Averages and standard error bars are shown (*n*=20)

To better understand the impact of recombination upon phylogenomic inference, we relaxed the simplifying assumption of uniform recombination rates across loci. We utilized two different approaches to simulate recombination hotspots along sequence alignments: one that was purely non-deterministic and the other which deterministically assigned the number of hotspots and their lengths. As shown in Fig. 3, the performance of the different methods was similar compared to our findings based on 8-taxon simulations with uniform recombination rate across loci: the LD-based methods were less accurate than the breakpoint-based methods, LD100 had comparable or reduced accuracy compared to LD1000, and IBIG had comparable or slightly worse accuracy compared to the other breakpoint-based methods. The topological accuracy of each method was generally comparable to its accuracy on the 8-taxon model condition with a uniform recombination rate across loci of 1000, with the exception of the LD1000 and breakpoint-based methods on the model condition with a purely non-deterministic hotspot model.

### Empirical study

**Fig. 4:**
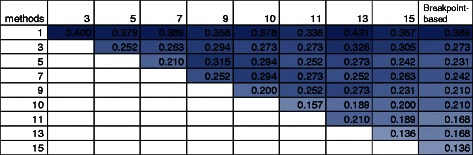
Pairwise comparison of species phylogenies inferred by breakpoint-based and LD-based methods on the empirical dataset. A species phylogeny was inferred for each mouse autosome using either the breakpoint-based method (“Breakpoint-based”) or an LD-based method. For the latter, sampled locus length varied between 1 SNP and at most 15 SNPs (which corresponds to an average genomic distance of around 100 kb, as shown in Additional file 1: Figure S5; each LD-based method is labeled by its sampled locus length (“1” through “15”). Pairwise topological comparisons are reported based upon average RF distance [26] across all mouse autosomes (*n*=19). Only upper triangular entries in the matrix are shown. Each cell is colored in a shade ranging from white to blue, corresponding to average normalized RF distance ranging from 0 to 1, respectively

No clear consensus exists regarding a fully resolved reference phylogeny for the empirical dataset in our study. Instead, we evaluated topological agreement among the methods themselves in terms of their inferred species phylogenies. Note that, unlike the simulation study, the empirical data were genotyped using the Mouse Genome Diversity microarray [29] which has relatively sparse sampling of sites across the mouse genome. Depending on the number of SNPs included in sampled loci used for LD-based analysis, the sampled locus length used in LD-based analyses varied from very short – spanning a single SNP – to very long – spanning almost 100 kb on average (Additional file 1: Figure S5). The latter is much longer than the 1 kb or 100 bp locus length of fine-scale sequence used in our simulation study. Figure 4 shows the pairwise topological agreement between LD-based and breakpoint-based methods, as measured by average normalized RF distance [26] across mouse autosomes. No matter the sampled locus length, the LD-based and breakpoint-based methods inferred species phylogenies that had average normalized RF distance of at least 0.136 and as much as 0.431. The latter is at the upper range observed in our simulation study (although the actual distance from any inferred phylogeny to the true phylogeny is unknown). The greatest topological agreement was observed between the breakpoint-based method and the LD-based methods with the longest sampled locus lengths (13 or 15 SNPs). As longer sampled locus lengths were used in the LD-based analyses, the topologies inferred by the LD-based and breakpoint-based methods became more similar. However, the topologies were still not in agreement even when the sampled locus length spanned an average of nearly 100 kb – almost two orders of magnitude longer than in the simulation study and much longer than the sampled locus length typically seen in phylogenomic studies. Among LD-based methods using different sampled locus lengths, the greatest pairwise topological agreement was observed using the longest lengths (more than 10 SNPs), and pairwise agreement tended to improve as sampled locus length increased.

## Discussion

Throughout our simulation study, we consistently found that the topological accuracy of the LD-based methods were worse than the breakpoint-based methods, including the IBIG method which uses a simple FGT-based approach for inferring recombination breakpoints. The topological accuracy of LD100 was comparable or worse than LD1000 on the smallest dataset sizes explored in our study (involving either 7 or 8 taxa), but the advantage of using longer locus length in LD-based pipelines was diminished on datasets with 15 or 25 taxa. The comparison of IBIG’s accuracy versus TBIG and TBTG suggests that the use of a simple technique for recombination breakpoint detection imposed little or no penalty in terms of topological accuracy. The performance advantage from utilizing inferred recombination breakpoints in lieu of LD-based preprocessing was observed across a range of recombination rates, dataset sizes, and models of recombination that incorporated both uniform recombination rate across loci as well as recombination hotspots. A word of caution is worth mentioning. The use of the FGT to detect recombination breakpoints may work well for our model conditions. However, as in the study of Lanier and Knowles [5] and other simulation studies, our simulation conditions make use of simplifying assumptions such as neutral evolution and small dataset sizes relative to current phylogenomic studies. It is unknown whether a simple breakpoint inference method will work well for more realistic evolutionary scenarios. More sophisticated alternatives may well be needed.

An increased recombination rate generally led to greater topological accuracy for both LD-based and breakpoint-based methods. Increasing recombination rate results in faster LD decay. Consequently, LD converges to background equilibrium at a shorter genomic distance, the LD-based methods utilize a shorter empirical LD decay cutoff, and LD-based preprocessing samples loci more finely across a sequence alignment. In general, less data loss yields more accuracy – a guideline based upon theory [30] and practice [5]. We attribute the relationship between recombination rate and the accuracy of the breakpoint-based methods to a related phenomenon. Increasing recombination rate resulted in more recombination breakpoints and therefore more gene trees (where a gene tree is inferred between each neighboring pair of breakpoints). The additional observations yielded more accuracy. We further attribute the impact of recombination rate upon IBIG’s accuracy to an additional factor: the additional breakpoints resulting from increasing recombination rate likely decreases the distance between an inferred breakpoint and the nearest true breakpoint, and the improved breakpoint inference accuracy propagates downstream during IBIG analysis. TBIG and TBTG make use of perfectly accurate recombination breakpoint and gene tree inputs, respectively; on the other hand, IBIG makes use of inferred recombination breakpoints that likely have high error. The comparison of TBIG and TBTG versus IBIG suggests that the breakpoint-based phylogenomic pipelines considered in this study are largely robust to inference error involving recombination breakpoints and/or local gene trees. Note an important distinction regarding the use of inferred recombination breakpoints. Consistent with [5], low to moderate recombination *within* a sampled locus doesn’t seem to impact topological accuracy to large extent; breakpoint inference error involving *inter*-locus recombination is similarly tolerable. Our findings were consistent across comparisons involving different levels of gene tree error (i.e., the comparison of LD1000 vs. LD100 and the comparison of IBIG vs. TBIG and TBTG) as well as an alternative pipeline that accounted for gene tree uncertainty (Additional file 1: Figures S9 – S11), suggesting that gene tree error was not a primary factor in our study.

In comparison to recombination rate, larger dataset sizes were seen to have a comparatively smaller impact upon topological accuracy. We note that the range of dataset sizes is relatively small by modern standards. Studies involving hundreds of genomes or more are becoming increasingly common [1]. We predict that dataset sizes of this scale or larger will have a stronger impact upon topological accuracy relative to the dataset sizes used in our study and others.

The greatest difference in accuracy of the breakpoint-based methods compared to the LD-based methods was observed on simulation conditions that incorporated an empirical phylogeny. Our interpretation of this finding is that the impact of recombination could be stronger for the types of topologies that form the Tree of Life, as opposed to random topologies typically generated by a Yule process. Traditional phylogenetic/phylogenomic inference pipelines fail to capture evolutionary factors which have had first-order effects upon the evolution of *Mus musculus* and sister species, including biogeography, natural selection, and co-evolution with human populations. We note that the same could be said for organisms that have been featured in other studies on recombination (e.g., humans and ancient hominins [31, 32], flowering plants [33], etc.). On model conditions with the highest recombination rate in our study, the breakpoint-based methods returned perfect accuracy and the LD-based methods were more accurate than on model conditions with lower recombination rates. We attribute this outcome to the differences between the empirical species tree and the random trees used elsewhere in our study. We further validated our findings using an additional set of simulations which incorporated the random model trees from [5]. Note that the random model trees generated in our study and in the study of [5] were produced using the same protocol; the only difference is that our study used 20 replicates and [5] used 10 replicates. Compared to the rest of our study, the performance of the LD-based and breakpoint-based methods were qualitatively similar, although the quantitative outcomes were somewhat different. We attribute the quantitative differences to the differing number of replicates used by the two studies.

In the empirical study, the comparative trends among the LD-based and breakpoint-based methods supported the performance findings from the simulation study. We observed a lack of topological agreement between the phylogeny inferred by the breakpoint-based method and any of the phylogenies inferred by the LD-based methods. In general, more loci and/or greater locus length resulted in greater topological agreement among the phylogenies inferred by different methods. As noted above, the empirical data is the outcome of a complex mix of disparate evolutionary forces. The simulation conditions explored in our study and others almost certainly fall short of capturing all relevant evolutionary processes. More effort is required to address this gap, particularly through the use of empirical data to drive methodological performance evaluation. The empirical data used in our study also had important limitations. Perhaps the biggest limitation is the array-based genotyping used to generate the data. The lack of fine-scale sequence data obscures our understanding of recombination in this empirical study. As a result, the average genomic distance spanned by a sampled locus (where three or more SNPs are included) was greater than in the simulation study by one to two orders of magnitude. Furthermore, empirical estimates of mouse recombination rates [34] suggest that, given the average genomic distance separating neighboring SNPs, each SNP should really serve as a separate locus. A more meaningful performance comparison based upon empirical data awaits the availability of fine-scale genomic sequence data (preferably whole genome sequences) from natural populations of different species. Fortunately, rapid advances in next-generation sequencing technology means that the availability of suitable datasets should be imminent.

## Conclusions

In this study, we have resolved Lanier and Knowles’s original question in the affirmative: indeed, recombination is a problem for widely used approaches to species tree analysis. While current phylogenomic methods for species tree inference may be largely robust to intra-locus recombination, the methodological assumption of free recombination *between* loci has major consequences upon phylogenetic inference accuracy, depending on the approach used to satisfy the assumption. The common LD-based sequence preprocessing used to accommodate this assumption is particularly problematic. We demonstrated that LD-based phylogenomic pipelines result in less accurate inference than breakpoint-based phylogenomic pipelines. We therefore recommend the use of computational techniques for explicitly inferring recombination breakpoints in lieu of LD-based sequence preprocessing. Although this substitution would seem to trade-off computational efficiency for accuracy, our study suggests that accurate species tree inference is possible even using simple and fast approaches for recombination breakpoint inference. The latter observation is in agreement with the findings of Lanier and Knowles; our study goes even further and amplifies their findings. Not only are breakpoint-based phylogenomic inference methods robust to violations of the assumption of zero intra-locus recombination, but also to breakpoint inference error and violations of the assumption of free recombination between loci.

Recombination is just one of several evolutionary processes that contribute to LD. Others include positive selection and population size variation. In the context of these other processes, LD-based preprocessing to satisfy the assumption of i.i.d. loci would likely have similar impacts on topological accuracy as those observed in our study. We need phylogenomic pipelines that explicitly account for these other processes and their impact on evolutionary histories. Note that recent modeling and methodological development to enable phylogenomic inference directly from sequence data are not immune either. For example, the SNAPP method introduced by [35] makes a similar assumption about its input. The question of how to extend these and other state-of-the-art approaches to account for recombination, natural selection, population size fluctuations, and other evolutionary processes alongside genetic drift and point mutations remains open.

The larger debate about how to choose suitable loci for species tree analysis also remains an open question. For example, [36] raised the question of whether concatenated analysis vs. coalescent-based analysis vs. “concatalescence”-based analysis (coalescent-based analysis of distant loci, where each locus concatenates multiple exons) is preferable. We note that all three are spanned by appropriate locus length and sampling interval choices. While Lanier and Knowles have shown that summary-based species tree inference is robust to longer “locus” length, it is natural to ask: how long is long enough, and how long is too long? In the limit, of course, increasing locus length approaches chromosome length and summary-based phylogenomic methods collapse into a concatenated analysis. And how shall we sample loci, regardless of length? Would a simple heuristic method suffice (e.g, a sliding window approach)? Or would a more principled approach be preferable? Our study provides only a partial resolution to these questions. For the evolutionary scenarios and simulation conditions explored in our study, our findings suggest that a phylogenomic inference pipeline which utilizes an approximation to recombination-free intervals based upon inferred recombination breakpoints is a reasonable option.

One finer distinction that must be underscored is the role of recombination in the context of phylogenomic inference: is it a nuisance, or is it in fact a missed opportunity? In our view, the question concerning the relative impact of recombination on phylogenomic inference accuracy is orthogonal to the potential phylogenomic signal offered by recombination. In theory, phylogenetic signal from recombination should be considered alongside phylogenetic signal produced by other evolutionary processes such as genetic drift, point mutations, and natural selection – all of which feature prominently in emerging methodological research. In practice, the relative contributions of each to genome evolution in different parts of the Tree of Life is unknown. We believe that an empirical evaluation requires methodologies which make use of the combination of signals from the different evolutionary processes at play. In contrast, none of the methods considered in our study nor in [5] make explicit use of signal from recombination for reconstructing phylogenetic relationships. It is possible that recombination alongside these other evolutionary processes mentioned above (but not generally explored together in simulation studies) will have combined impact on topological accuracy that is greater than the sum of individual effects. Rather than ignoring recombination as negligible noise, we encourage the research community to revisit its role in species tree inference.

## Additional file

Additional file 1 Appendix with Supplementary Material. (PDF 506 kb)
