# Supplementary material for: A performance study of the impact of recombination on species tree analysis
Source: BMC Genomics. 2016 Nov 11;17(Suppl 10):785. doi: 10.1186/s12864-016-3104-5 (PMC5123380; doi:10.1186/s12864-016-3104-5)
Supplement: Supplementary file 1 — Appendix with Supplementary Material. (PDF 506 kb) [file 12864_2016_3104_MOESM1_ESM.pdf]

Appendix with Supplementary Materials

Supplementary Methods

Commands and Software Options

Model trees were generated using the following Mesquite [1] options:

1. file
2. new file
3. input taxa number
4. taxa&trees
5. save trees to file from simulated trees
6. uniform distribution
7. input total tree depth

The empirical tree used as an alternative model tree in the simulation study was:

```
(((((Mus musculus:1.7,(Mus spicilegus:1.0,Mus macedonicus:1.0):0.7):0.8,
(Mus fragilicauda:2.1,Mus famulus:2.1):0.4):0.5,
(Mus caroli:2.5,Mus cookii:2.5):0.5):1.2,Pyromys:4.2):0.8,Nannomys:5.0);
```

The ms [2] command used to perform coalescent simulations given a model tree was ms <n> 1 -t 10000 -r <ρ> 10000000 -I nt 1 1 1 ... -ej <coalhis> -T, where <n> is the number of taxa in the species tree and <coalhis> is the coalescent history.

Gene trees were estimated using the following FastTree [3, 4] command: ./FastTree -nt -gtr -nosupport <input sequence file name>.

We used the following command to perform ASTRAL-II [5, 6] analyses: java -jar astral -i <name of input file containing gene trees> -o <name of output file containing estimated species tree>.

The msHOT [7] command used in our study was ./msHOT <nt> 1 -t 0.38 -r ρ 10000000 -v <nh> <n_{i1}> <n_{i2}> <s_i> -I <nt> 1 1 1 ... -ej <coalhis> -T where <nt> is the number of taxa in the species tree, <coalhis> is the coalescent history, <n_{i1}> is the start position of the ith hotspot, <n_{i2}> is the end position of the ith hotspot, and s_i is the factor that determines the hotspot vs. background recombination rate ratio for the ith hotspot.

PhyloNet [8] calculations were run using the command java -jar PhyloNet.jar <nexfile name>, where <nexfile name> was the filename for a Nexus input file that contained topological distance calculation commands.

All datasets and software are publicly available under an open copy-left license at https://gitlab.msu.edu/liulab/impact-of-recombination-on-phylogenetic-inference.materials.

Supplementary Results

Simulation study

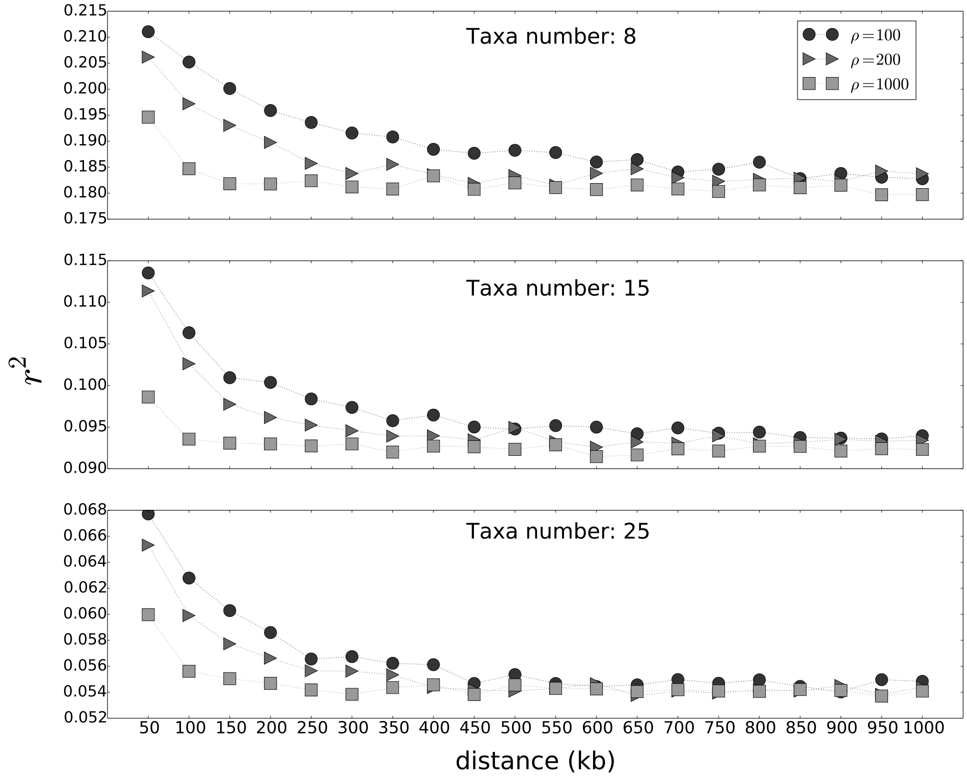

Supplementary Figure S1: **LD decay plot of simulated datasets.** LD is quantified using r^2. Each data point in the plot is the average r^2 of all the pairwise loci between which the distances ranges from α to β. β is the point's x-axis index, α is the point's left neighbor's x-axis index. The result LD decay patterns of datasets with different recombination rates in 8-taxon, 15-taxon, and 25-taxon model conditions are shown.

Supplementary Table S1: **LD decay cut-off values used in LD-based methods estimating simulated model phylogenies.** The cut-off values of the data sets simulated with different recombination rates in 8-taxon, 15-taxon, and 25-taxon model conditions were decided according to LD decay plots in Figure S1.

Model trees	ρ	cut-off value (kb)
8-taxon	100	800
8-taxon	200	500
8-taxon	1000	200
15-taxon	100	800
15-taxon	200	500
15-taxon	1000	200
25-taxon	100	800
25-taxon	200	500
25-taxon	1000	200

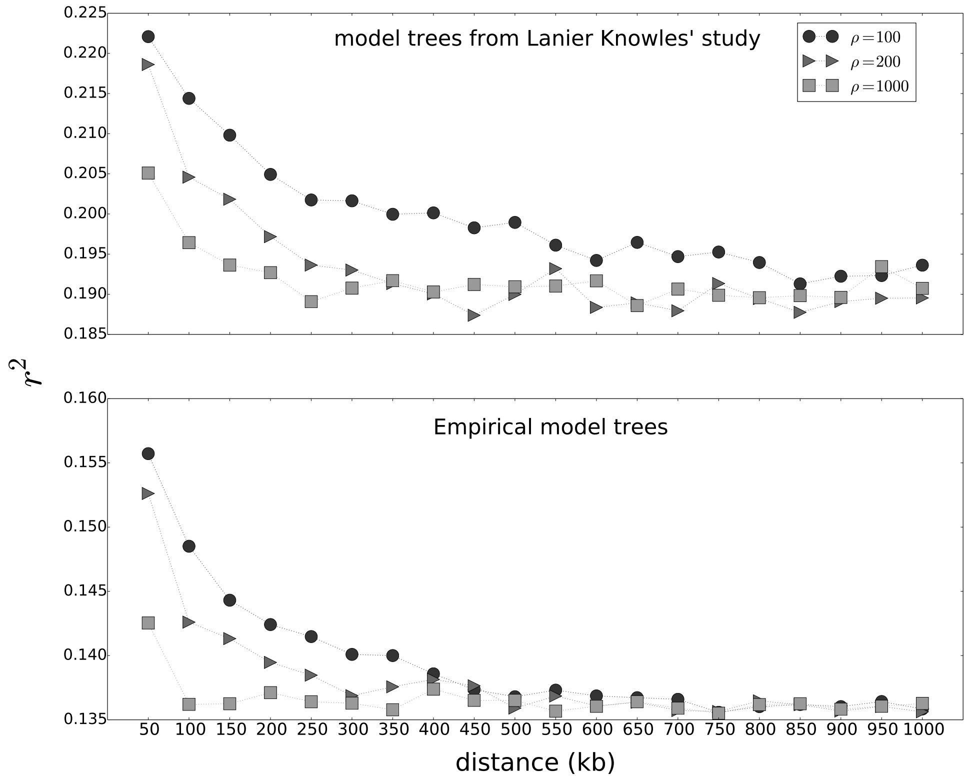

Supplementary Figure S2: **LD decay plots for datasets simulated using alternate model phylogenies.** LD is quantified using r^2. Each data point in the plot is the average r^2 of all the pairwise loci between which the distances ranges from α to β. β is the point's x-axis index, α is the point's left neighbor's x-axis index. The upper panel shows results for simulations using the model phylogenies from the study of Lanier and Knowles [9]. In the lower panel, results are shown for simulations using a model phylogeny that is a consensus estimate based upon multiple empirical studies (see Methods). In each panel, the result LD decay patterns of datasets simulated with different recombination rates are shown.

Supplementary Table S2: **LD decay cut-off values used in LD-based methods estimating alternate model phylogenies.** The cut-off values of data sets simulated with different recombination rates in the alternate model conditions were decided according to LD decay plots in Figure S2.

Model trees	ρ	cut-off value (kb)
Lanier&Knowles' study	100	850
Lanier&Knowles' study	200	450
Lanier&Knowles' study	1000	250
Empirical phylogenies	100	750
Empirical phylogenies	200	500
Empirical phylogenies	1000	100

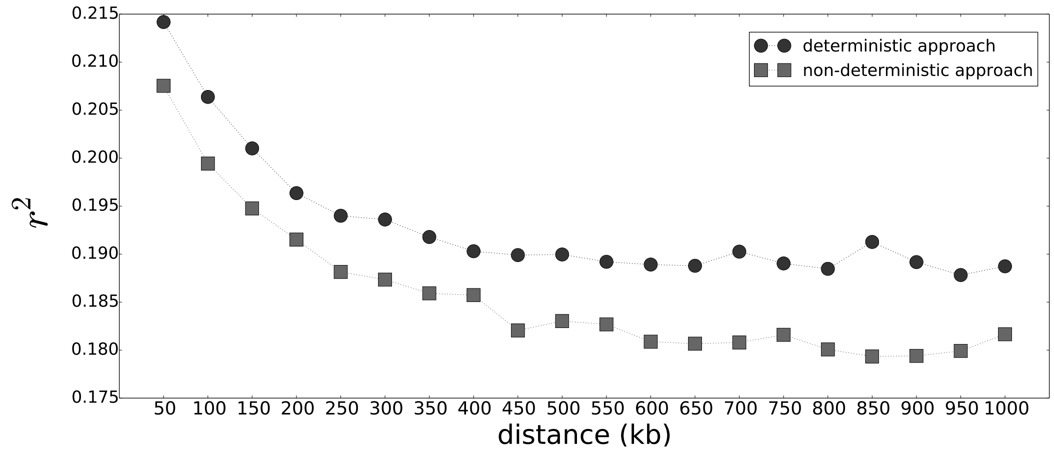

Supplementary Figure S3: **LD decay plot for datasets simulated with recombination hotspots.** LD is quantified by r^2. Each data point in the plot is the average r^2 of all the pairwise loci between which the distances ranges from α to β. β is the point's x-axis index, α is the point's left neighbor's x-axis index. Recombination hotspots were sampled using either a deterministic or non-deterministic approach (see Methods). The LD decay cut-off value used by the LD-based methods in both panels was 800 kb.

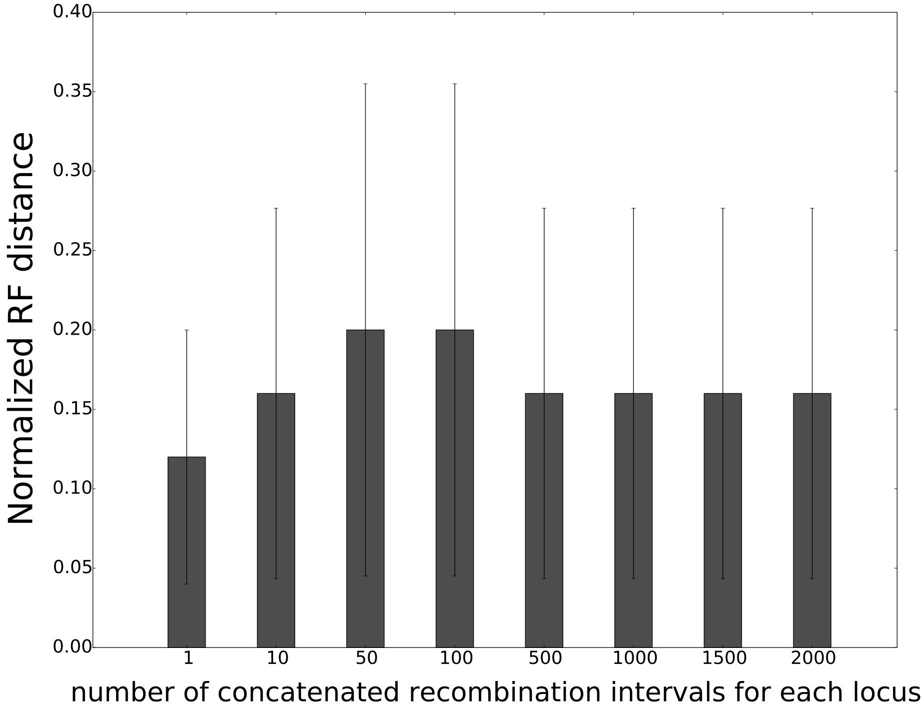

Supplementary Figure S4: **Impact of number of concatenated recombination free intervals for each locus.** Results are shown for the 8-taxon model condition with $\rho = 100$. The x-axis is the number of concatenated recombination free intervals for each locus used in IBIG analysis. Note that, for readability, the x-axis tics are not plotted to scale. The average normalized RF distance of 5 replicates are shown.

Empirical study

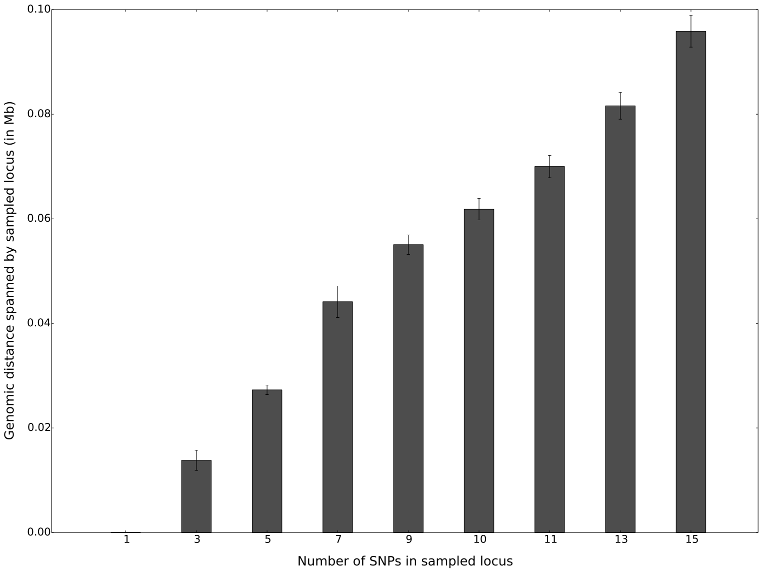

Supplementary Figure S5: **Genomic distance spanned by sampled loci used in LD-based analysis.** Each LD-based analysis utilized a sampled locus length that varied between 1 SNP and at most 15 SNPs. The average genomic distance spanned by sampled loci are shown for each LD-based analysis.

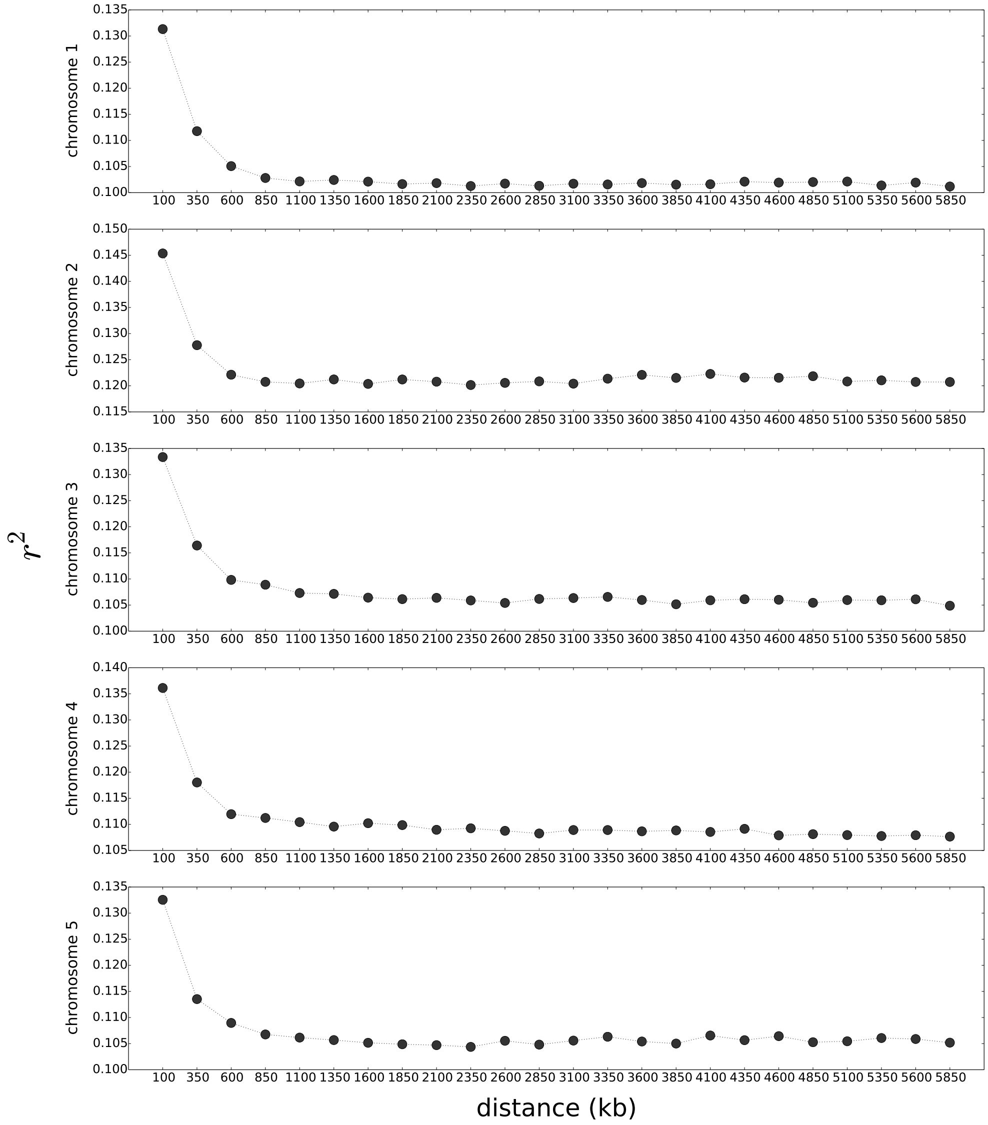

Supplementary Figure S6: **LD decay plots for chromosomes 1 through 5.** LD is quantified using r^2. Each data point in the plot is the average r^2 of all the pairwise loci between which the distances ranges from α to β. β is the point's x-axis index, α is the point's left neighbor's x-axis index. Each panel shows results for one chromosome.

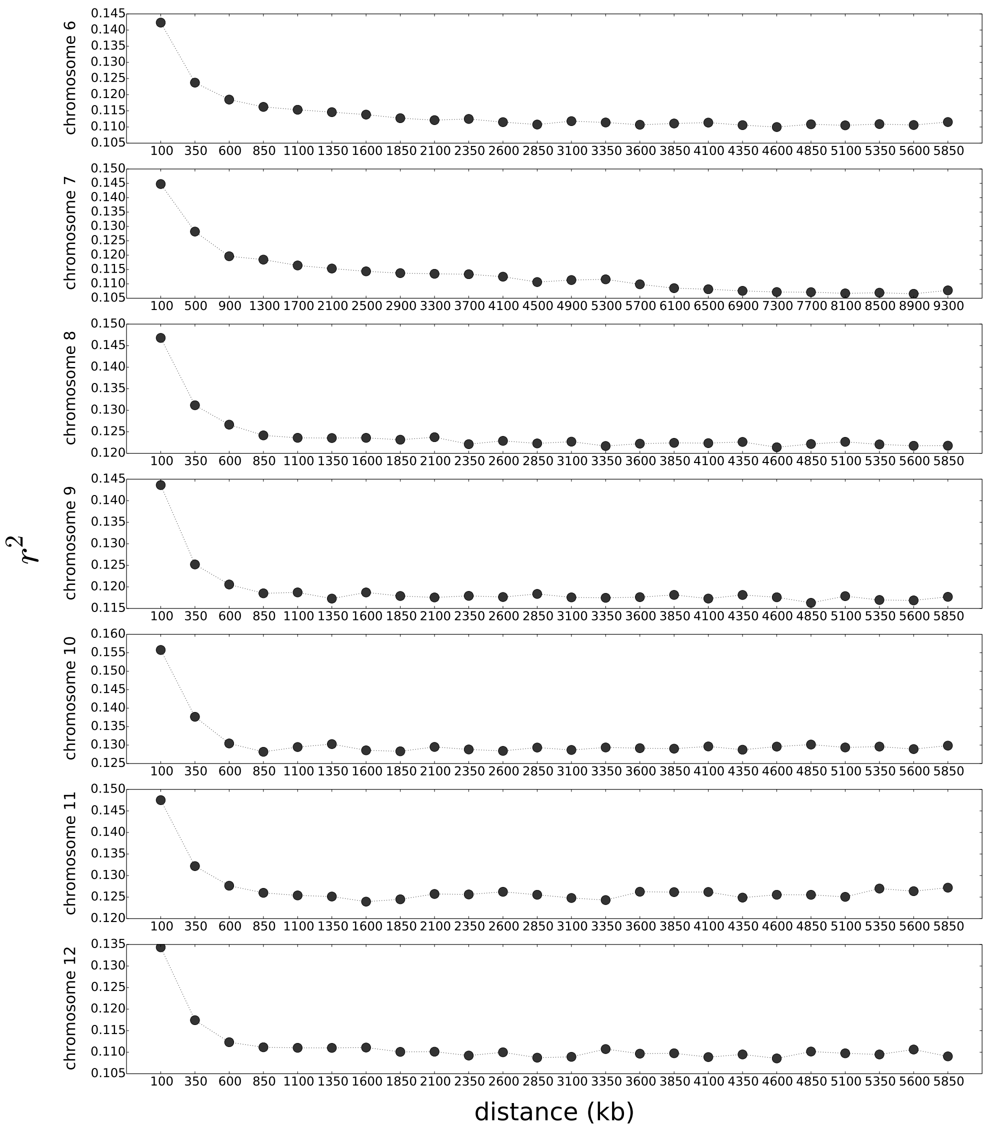

Supplementary Figure S7: **LD decay plots for chromosomes 6 through 12.** Figure layout and description are otherwise identical to Supplementary Figure S6.

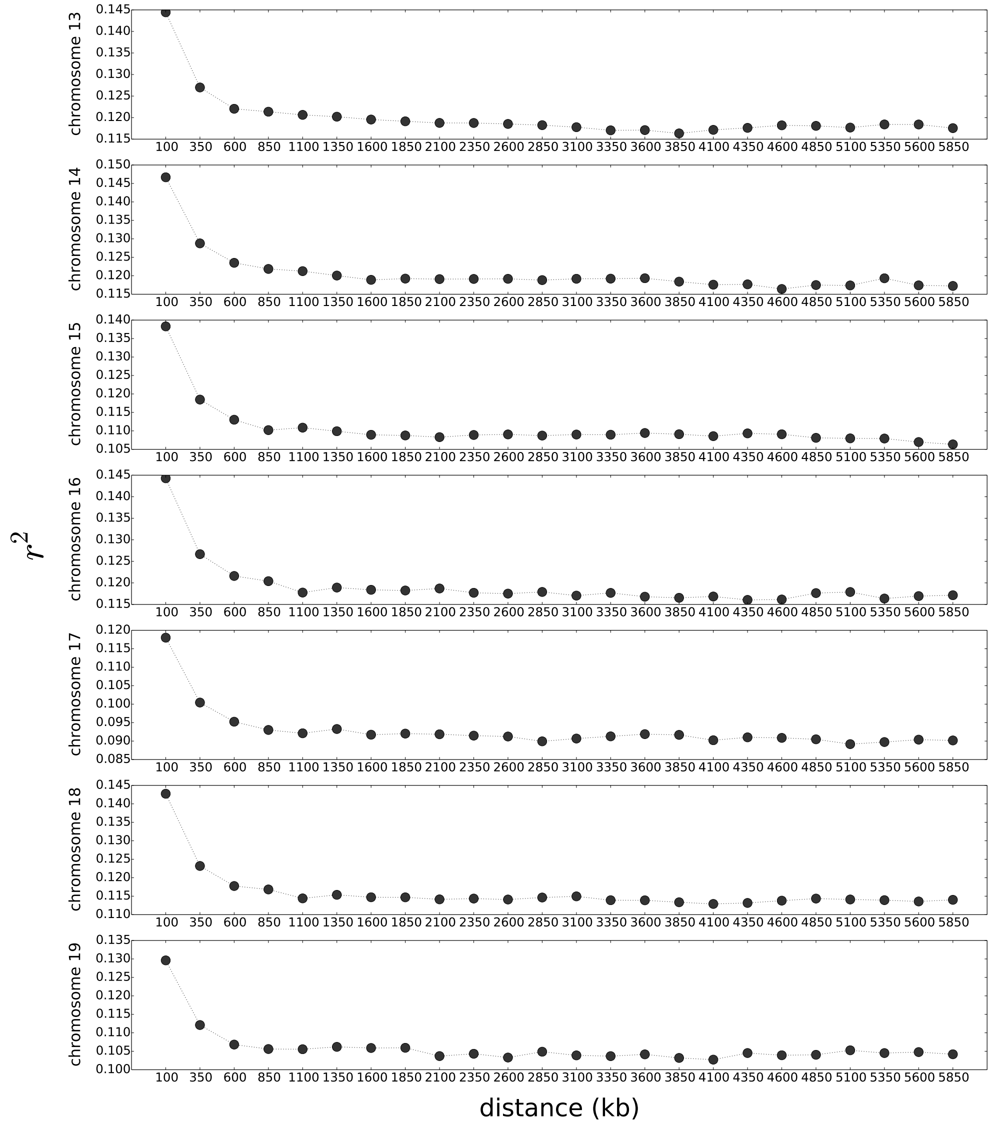

Supplementary Figure S8: **LD decay plot for chromosomes 13 through 19.** Figure layout and description are otherwise identical to Supplementary Figure S6.

Supplementary Table S3: **LD decay cut-off values used in LD-based methods to preprocess datasets of each chromosome.** The cut-off values were chosen according to the Figures S6, S7 and S8.

chromosome	cut-off value (Mb)	chromosome index	cut-off value (Mb)
1	2	11	1
2	1	12	1
3	2	13	3
4	2	14	2
5	1	15	2
6	3	16	3
7	7	17	2
8	2	18	1
9	1	19	2
10	1		

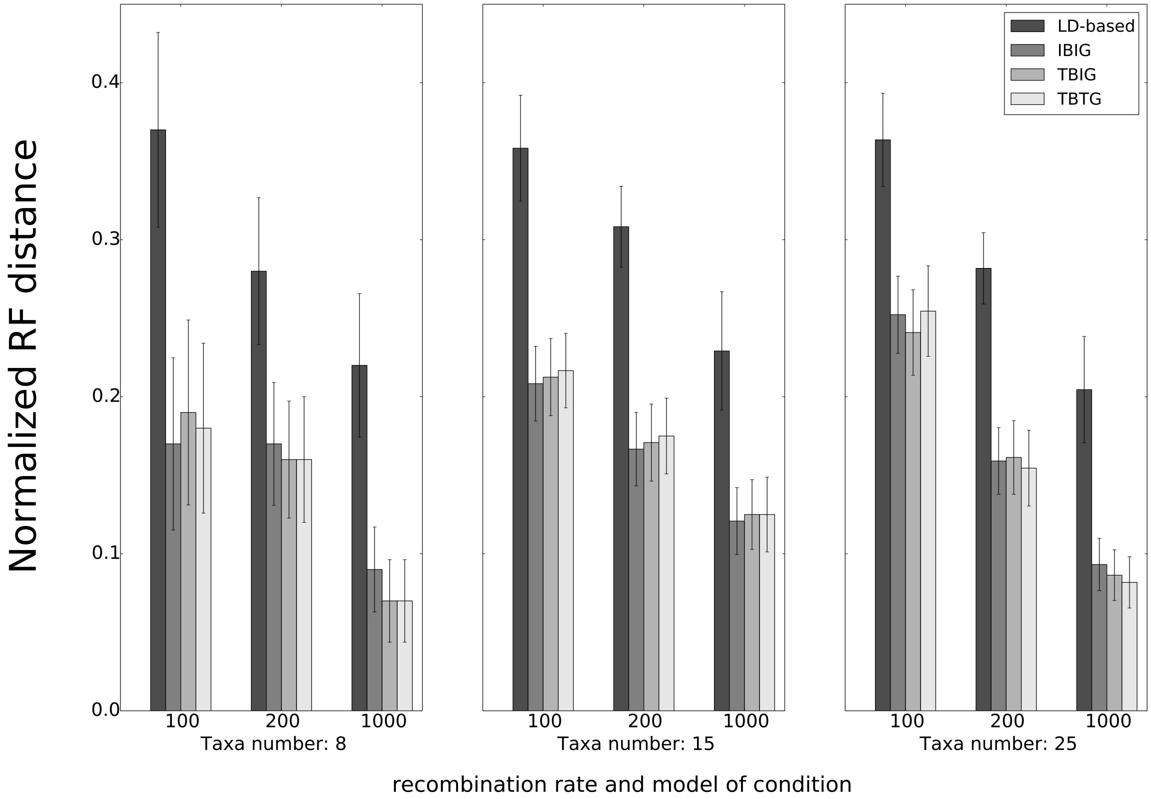

Supplementary Figure S9: **Topological comparison of breakpoint-based methods vs. an LD-based method that accounts for gene tree uncertainty, where gene tree edges with support less than 0.1 were discarded.** The breakpoint-based methods are identical to those used elsewhere in the study. The method labeled "LD-based" was a modified version of the LD100 method which accounted for gene tree uncertainty using the same procedure from [5]. Specifically, we used LD-based preprocessing to obtain a set of loci with locus length of 100 bp, FastTree [3, 4] was used to infer a gene tree for each locus along with estimated support values for all gene tree edges, gene tree edges with support less than 0.1 were collapsed, and the (possibly non-binary) gene trees were used as input to coalescent-based species tree inference using ASTRAL-II [5, 6].

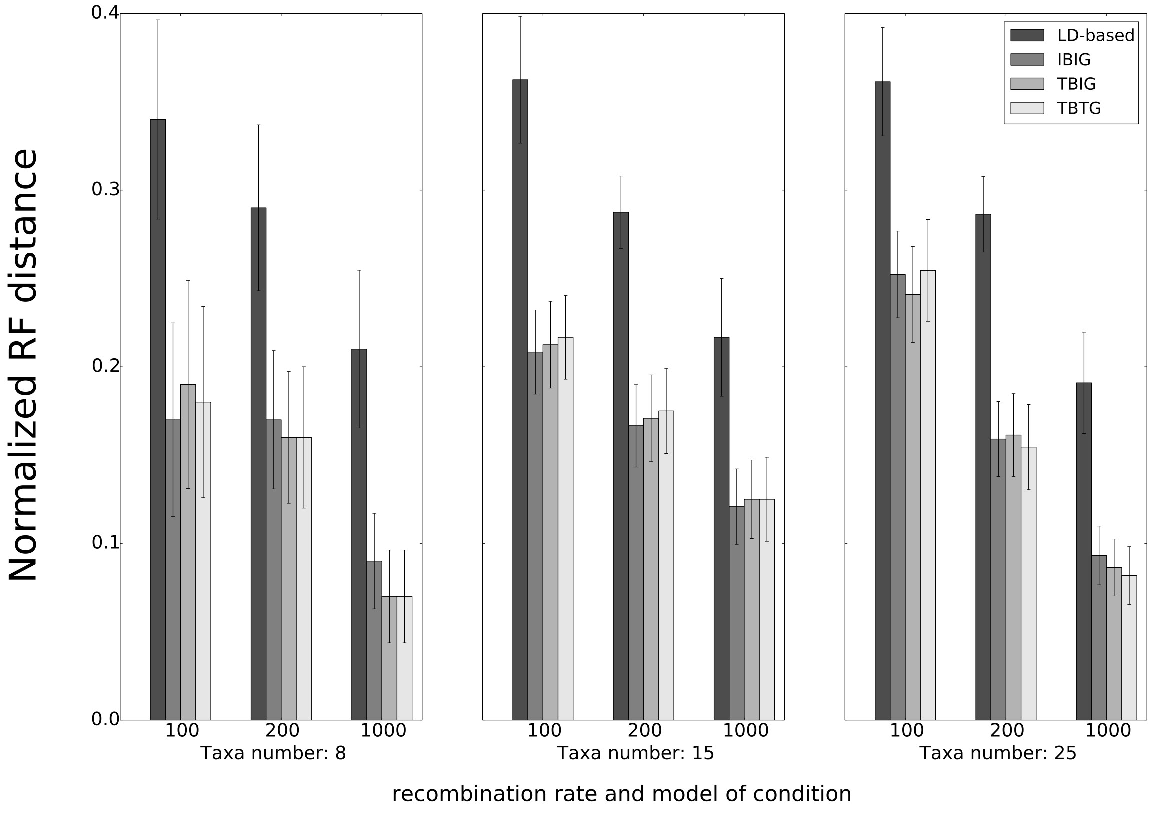

Supplementary Figure S10: **Topological comparison of breakpoint-based methods vs. an LD-based method that accounts for gene tree uncertainty, where gene tree edges with support less than 0.33 were discarded.** Figure layout and description are otherwise identical to Supplementary Figure S9.

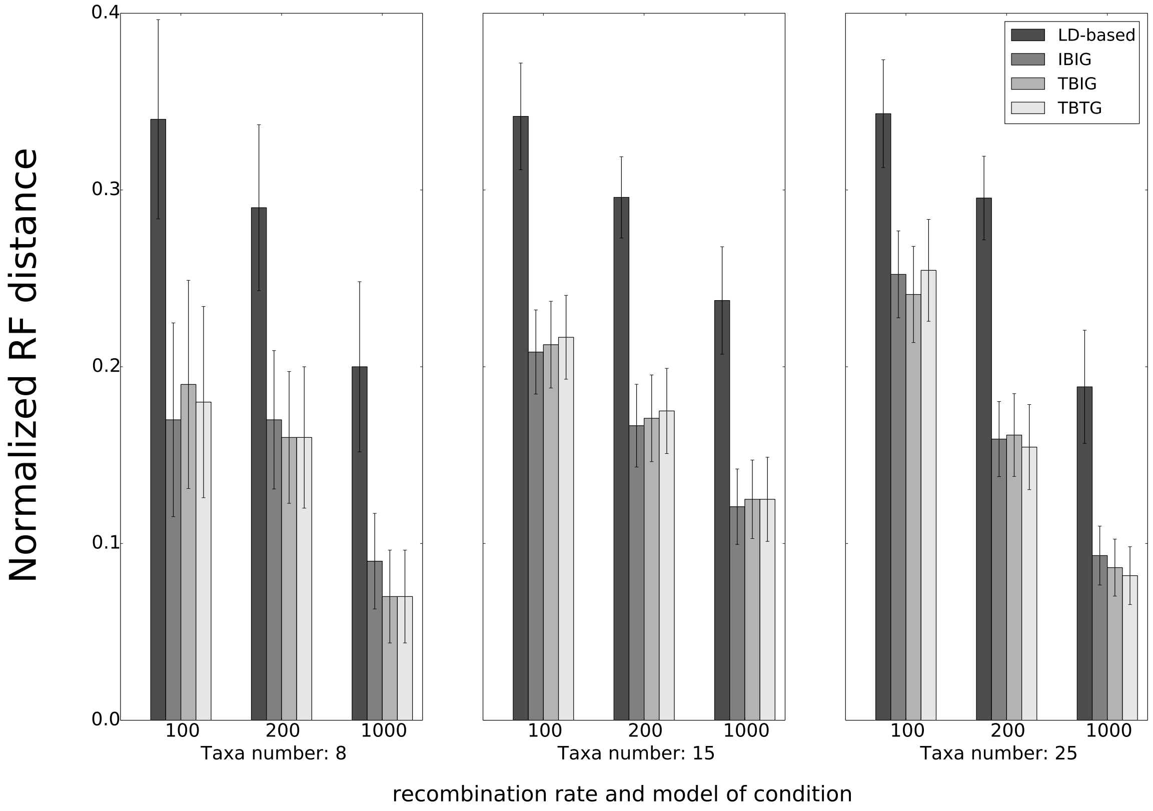

Supplementary Figure S11: **Topological comparison of breakpoint-based methods vs. an LD-based method that accounts for gene tree uncertainty, where gene tree edges with support less than 0.5 were discarded.** Figure layout and description are otherwise identical to Supplementary Figure S9.

References

[1] Maddison, W.P., Maddison, D.R.: Mesquite: a modular system for evolutionary analysis, version 1.05 (2004)

[2] Hudson, R.R.: Generating samples under a Wright-Fisher neutral model of genetic variation. Bioinformatics **18**(2), 337–338 (2002). doi:10.1093/bioinformatics/18.2.337. http://bioinformatics.oxfordjournals.org/content/18/2/337.full.pdf+html

[3] Price, M., Dehal, P., Arkin, A.: FastTree 2 - approximately maximum-likelihood trees for large alignments. PLoS ONE **5**(3), 9490 (2010). doi:10.1371/journal.pone.0009490

[4] Price, M., Dehal, P., Arkin, A.: FastTree: Computing large minimum evolution trees with profiles instead of a distance matrix. Molecular Biology and Evolution **26**(7), 1641–1650 (2009). doi:10.1093/molbev/msp077. http://mbe.oxfordjournals.org/content/26/7/1641.full.pdf+html

[5] Mirarab, S., Warnow, T.: ASTRAL-II: coalescent-based species tree estimation with many hundreds of taxa and thousands of genes. Bioinformatics **31**(12), 44–52 (2015)

[6] Mirarab, S., Reaz, R., Bayzid, M.S., Zimmermann, T., Swenson, M.S., Warnow, T.: ASTRAL: genome-scale coalescent-based species tree estimation. Bioinformatics **30**(17), 541–548 (2014)

[7] Hellenthal, G., Stephens, M.: msHOT: modifying Hudson's ms simulator to incorporate crossover and gene conversion hotspots. Bioinformatics **23**(4), 520–521 (2007)

[8] Than, C., Ruths, D., Nakhleh, L.: PhyloNet: a software package for analyzing and reconstructing reticulate evolutionary relationships. BMC Bioinformatics **9**(1), 322 (2008)

[9] Lanier, H.C., Knowles, L.L.: Is recombination a problem for species-tree analyses? Systematic Biology **61**(4), 691–701 (2012)
